# Identifying the Genome-Wide Sequence Variations and Developing New Molecular Markers for Genetics Research by Re-Sequencing a Landrace Cultivar of Foxtail Millet

**DOI:** 10.1371/journal.pone.0073514

**Published:** 2013-09-10

**Authors:** Hui Bai, Yinghao Cao, Jianzhang Quan, Li Dong, Zhiyong Li, Yanbin Zhu, Lihuang Zhu, Zhiping Dong, Dayong Li

**Affiliations:** 1 Institute of Millet Crops, Hebei Academy of Agriculture and Forestry Sciences, Shijiazhuang, Hebei, China; 2 National Foxtail Millet Improvement Center, Shijiazhuang, Hebei, China; 3 State Key Laboratory of Plant Genomics and National Center for Plant Gene Research, Institute of Genetics and Developmental Biology, Chinese Academy of Sciences, Beijing, China; 4 College of Life Sciences, Agricultural University of Hebei, Baoding, Hebei, China; 5 Minor Cereal Crops Laboratory of Hebei Province, Shijiazhuang, Hebei, China; Ben-Gurion University, Israel

## Abstract

Foxtail millet ( *Setaria* *italica* ) is a drought-resistant, barren-tolerant grain crop and forage. Currently, it has become a new model plant for cereal crops and biofuel grasses. Although two reference genome sequences were released recently, comparative genomics research on foxtail millet is still in its infancy. Using the Solexa sequencing technology, we performed genome re-sequencing on one important foxtail millet Landrace, Shi-Li-Xiang (SLX). Compared with the two reference genome sequences, the following genetic variation patterns were identified: 762,082 SNPs, 26,802 insertion/deletion polymorphisms of 1 to 5 bp in length (indels), and 10,109 structural variations (SVs) between SLX and Yugu1 genomes; 915,434 SNPs, 28,546 indels and 12,968 SVs between SLX and Zhang gu genomes. Furthermore, based on the Yugu1 genome annotation, we found out that ~ 40% SNPs resided in genes containing NB-ARC domain, protein kinase or leucine-rich repeats, which had higher non-synonymous to synonymous SNPs ratios than average, suggesting that the diversification of plant disease resistance proteins might be caused by pathogen pressure. In addition, out of the polymorphisms identified between SLX and Yugu1, 465 SNPs and 146 SVs were validated with more than 90% accuracy, which could be used as DNA markers for whole-genome genotyping and marker-assisted breeding. Here, we also represented an example of fine mapping and identifying a *waxy* locus in SLX using these newly developed DNA markers. This work provided important information that will allow a deeper understanding of the foxtail millet genome and will be helpful for dissecting the genetic basis of important traits in foxtail millet.

## Introduction

Foxtail millet [ *Setaria* *italica* (L.) Beauv.] is an important minor cereal crop, originated from Northern China with more than 8,700 years in cultivation history [1–3]. Because of its remarkable characteristics of drought-resistance and barren-tolerance [4,5], foxtail millet becomes much more important for agriculture in the arid and barren environment [6–8]. In addition, foxtail millet is a self-fertilizing and C_4_ panicoid crop with a small diploid genome [9]. With the release of two draft genome sequences of foxtail millet cultivars, Yugu1 and Zhang gu [10,11], it is rapidly becoming another tractable experimental model for the functional genomics research on cereal crops and biofuel grasses [9].

The completion of the two reference genome sequences of foxtail millet is a great advance for the comparative and functional genomics studies on the grass family, and the resulting genetic and genomic data are an invaluable resource for biology research and genetics improvement of foxtail millet [10–12]. Compared with other model plants and important crops, the genomics study on foxtail millet is still in its infancy. Therefore, it is still essential to get more genome information for foxtail millet.

The application of the next-generation high-throughput DNA sequencing technologies has made re-sequencing of various crops possible, accelerating the identification of genome-wide patterns of genetic variation in crop populations, the investigation of the origin and domestication processes of cultivated crops by population genetics analysis, and the further exploration of relations between genotypes and phenotypes in crops by genotyping and genome-wide association studies (GWAS), which greatly promotes crops genomics and genetics research [12–24]. For example, Huang et al. [25] used low-coverage re-sequencing of genomes of a panel of 517 rice landraces, constructed a high-density haplotype map of the rice genome and found 80 loci associated with 14 agronomic traits. Therefore, the reference genomes and sequences by re-sequencing of wild and cultivated foxtail millet varieties will lay a solid foundation for functional and comparative genomics studies on foxtail millet [24].

It is well known that germ plasm resources have been successfully used in traditional breeding efforts to improve crop plants [26–28]. There are abundant germ plasm resources of foxtail millet around the world, which are rich breeding materials [9]. In this study, the Solexa sequencing technology and the Genome Analyzer ii (GA ii) were employed to re-sequence the genome of a foxtail millet landrace, Shi-Li-Xiang (SLX) and to analyze its genetic structures. We developed a great deal of effective DNA markers for exploring agronomic traits-related genes by comparing the obtained SLX sequences with those of the two published reference genomes. In addition, as an example of application of our newly developed markers, we mapped a *waxy* locus in SLX with assistance of these markers. This work provides a rich tag library for future genetic studies and molecular breeding (such as rust resistance, waxy trait and indicative character in breeding) of foxtail millet and its related species.

## Results and Discussion

### In silico mapping of re-sequencing reads to the reference foxtail millet genomes

**Figure 1:**
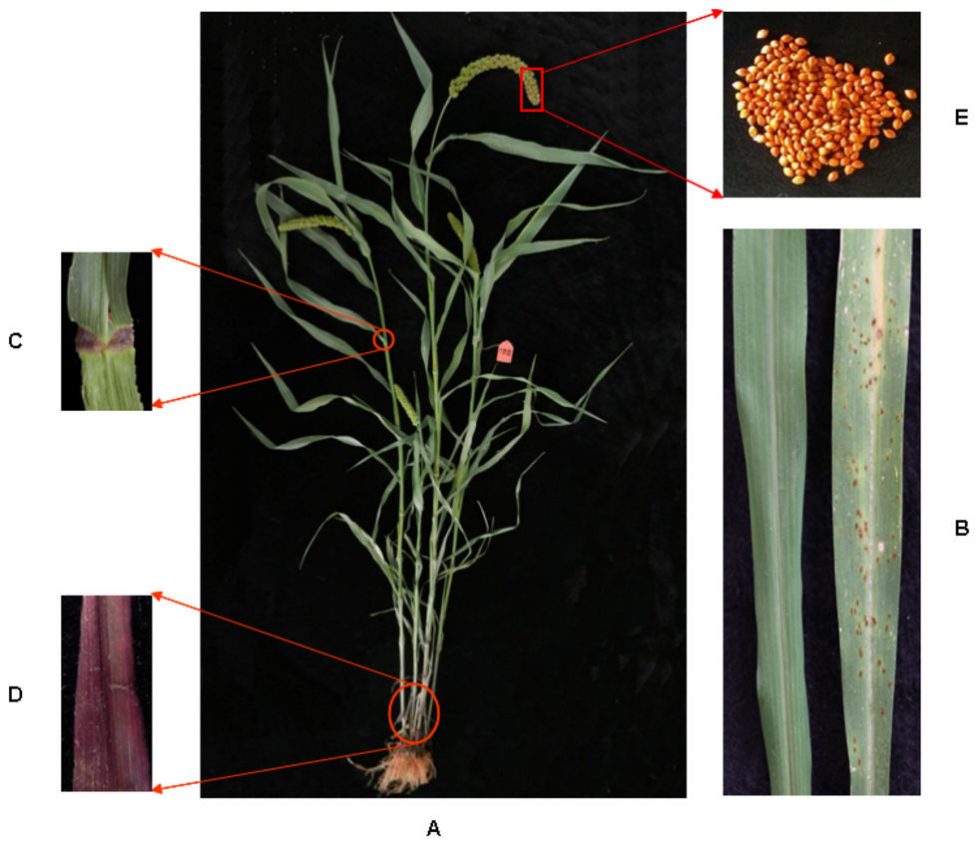
Characteristics of the foxtail millet landrace, Shi-Li-Xiang (SLX). A, Gross morphology of SLX at reproductive phase (2 months old). B, SLX is highly resistant against rust disease. The susceptible cultivar Yugu1 is shown as a control. The leaf phenotypes of SLX (highly resistant, left) and Yugu1 (highly susceptible, right) at 14 days after inoculated with the *Uromycessetariae-italicae* Yoshino strains 93-5. C, Purple pulvinus. D, Purple leaf sheath. E, Red seed coat.

We previously identified and characterized cultivars and landraces with disease resistance from our collection of foxtail millet [ *Setaria* *italica* (L.) Beauv.] germ plasm resources (unpublished data). One of these landraces, Shi-Li-Xiang (SLX), showed highly resistance against rust disease during whole growth period [29]. In addition to disease resistance, SLX showed other excellent agronomic traits, such as waxy trait for taste, purple pulvinus and leaf sheath as indicative phenotypes in breeding, and red seed coat (Figure 1). Meanwhile, it also displayed some negative agronomic characters, such as more tillers and lower yield. Taken together, SLX is a special resource for foxtail millet breeding improvement and genetic study. For all these reasons, SLX is selected for re-sequencing in this study.

**Figure 2:**
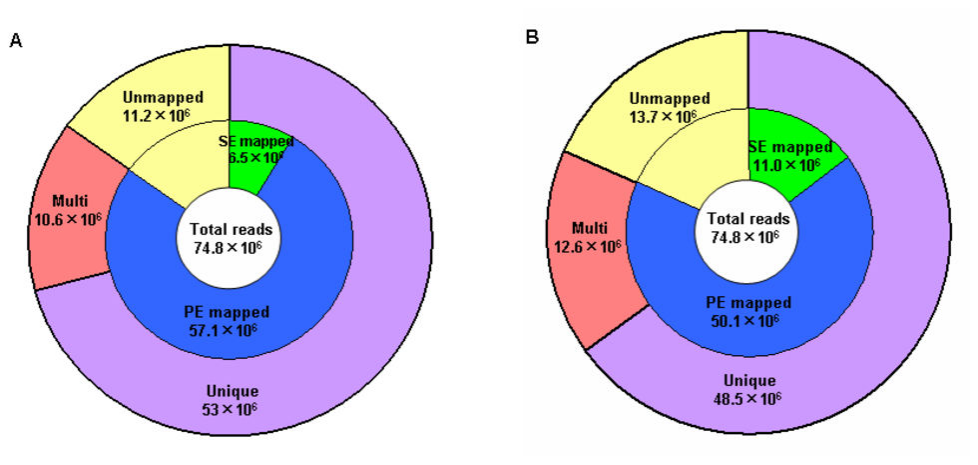
Classification of SLX reads mapped onto the Yugu1 and Zhang gu genomes, respectively. A, SLX aligned with Yugu1 genome. B, SLX aligned with Zhang gu genome. The total number of mapped reads is in the center circle. The numbers of pair-end and single-end reads mapped onto chromosome and unmapped reads are shown in the middle circle. The outer circle represents unique or multiple mapping on chromosomes.

Whole-genome sequencing was performed on the genomic DNA sample extracted from SLX by using the Solexa sequencing technology and the Genome Analyzer ii (GA ii). A total of 74,792,844 short reads of 76 nucleotides (74.8×10^6^ reads henceforth) were generated. Using Burrows-Wheeler alignment (BWA) tool software [30], 63.6×10^6^ and 61.1×10^6^ of the obtained reads were successfully mapped onto the two reference genomes, Yugu1 (under the accession AGNK00000000 at DDBJ/EMBL/GenBank) and Zhang gu (ftp://ftp.genomics.org.cn/pub/Foxtail_millet), respectively (Table 1) [10,11]. The average sequencing depth was approximately 11-fold, and the resulting consensus sequences covered approximately 93% of the two reference genomes (Table 1). Among them, 57.1×10^6^ pair-end (PE) reads and 6.5×10^6^ single-end (SE) reads were mapped to chromosomes corresponding to 405.7 Mb of the Yugu1 genome, while a total of 50.1 ×10^6^ PE reads and 11.0 ×10^6^ SE reads were mapped to chromosomes corresponding to 400.1 Mb of the Zhang gu genome (Table 1 and Figure 2). Although both of the sequenced reads from SLX aligned with the two reference genomes were above 80%, the PE reads from SLX aligned with Yugu1 were more than the ones aligned with Zhang gu. Furthermore, a total of 53×10^6^ and 48.5×10^6^ reads were uniquely mapped to Yugu1 and Zhang gu chromosomes, respectively, and the remaining 10.6×10^6^ and 12.6×10^6^ reads were mapped to multiple locations (Figure 2). Our results suggested that the genetic relationship between SLX and Yugu1 might be closer than that between SLX and Zhang gu.

**Table 1:** Summary of original sequencing data of SLX.

**Sample**	**Total_reads**	**PE (%**)	**SE (%**)	**Total_map (%**)	**Unique_map (%**)	**Multiple_map (%**)	**Identity (%**)	**Depth**	**Cover_ratio (%**)
**SLX_Yugu1**	74,792,844	76.40	8.63	85.03	83.40	16.60	99.21	11.19	93.51
**SLX_Zhang gu**	74,792,844	67.02	14.65	81.67	79.44	20.56	98.97	11.36	93.43

### Detection and distribution of variations

According to the alignment result between SLX and the two reference genomes, genome-wide single-nucleotide polymorphisms (SNPs) were examined via the samtools software [31], and a total of 762,082 and 915,434 SNPs were detected between SLX and Yugu1, and between SLX and Zhang gu, respectively. Obviously the number of the detected SNPs in the latter comparison is more than that in the former. Also, genome-wide insertion/deletion polymorphisms of 1- to 5-bp in length (InDels) and structural variations (SVs) were examined via both pindel [32] and breakdancer [33] softwares. 26,802 InDels and 10,109 SVs were yielded from SLX aligned with Yugu1, and 28,546 InDels and 12,968 SVs were yielded from SLX aligned with Zhang gu. The overall genome diversity between SLX and Yugu1 was much lower than that between SLX and Zhang gu, indicating the possible closer relationship between SLX and Yugu1. Previous studies on rice show that the total number of SNPs, InDels and SVs which have been detected varies across different chromosomes [34,35]. Among the variations detected between SLX and Yugu1, the highest number of SNPs (123,932) was observed in chromosome 8, while chromosome 2 had the highest number of InDels (3,843) and SVs (1,473), and the lowest numbers of SNPs (42,772), InDels (1,762) and SVs (732) were observed in chromosome 1 (Table 2). However, among the variations detected between SLX and Zhang gu, the highest number of SNPs, InDels and SVs (176,121,4,401,1,829) was observed in chromosome 8, and the chromosome 4 had the lowest number of them (60,809, 1,656 and 881) (Table 2).

**Table 2:** Variations detected for SLX.

**Chromosome**	**SNPs**			**Indels**			**SVs**	
	**SLX vs Yugu1**	**SLX vs Zhang gu**		**SLX vs Yugu1**	**SLX vs Zhang gu**		**SLX vs Yugu1**	**SLX vs Zhang gu**
**Chr01**	42772 (1014.9)	70103 (1661.6)		1762 (41.8)	2422 (57.4)		732 (17.3)	1101 (26.1)
**Chr02**	104857 (2131.2)	118018 (2388.0)		3843 (78.1)	3983 (80.6)		1473 (29.9)	1744 (35.3)
**Chr03**	83862 (1655.6)	104183 (2199.7)		3479 (68.7)	3455 (72.9)		1281 (25.3)	1585 (33.5)
**Chr04**	54686 (1353.3)	60809 (1517.4)		1910 (47.3)	1656 (41.3)		729 (18.0)	881 (22.0)
**Chr05**	60386 (1277.9)	84822 (1750.1)		2390 (50.6)	2926 (60.4)		906 (19.2)	1400 (28.9)
**Chr06**	60912 (1691.3)	95618 (2561.7)		2273 (63.1)	2957 (79.2)		857 (23.8)	1219 (32.7)
**Chr07**	116946 (3251.7)	112190 (3118.9)		3668 (102.0)	3814 (106.0)		1340 (37.3)	1612 (44.8)
**Chr08**	123932 (3045.8)	176121 (4058.7)		3785 (93.0)	4401 (101.4)		1459 (35.9)	1829 (42.1)
**Chr09**	113729 (1928.6)	93570 (1673.1)		3692 (62.6)	2932 (52.4)		1332 (22.6)	1597 (28.5)
**Total**	762082 (1899.0)	915434 (2287.8)		26802 (66.8)	28546 (71.3)		10109 (25.2)	12968 (32.4)

**Figure 3:**
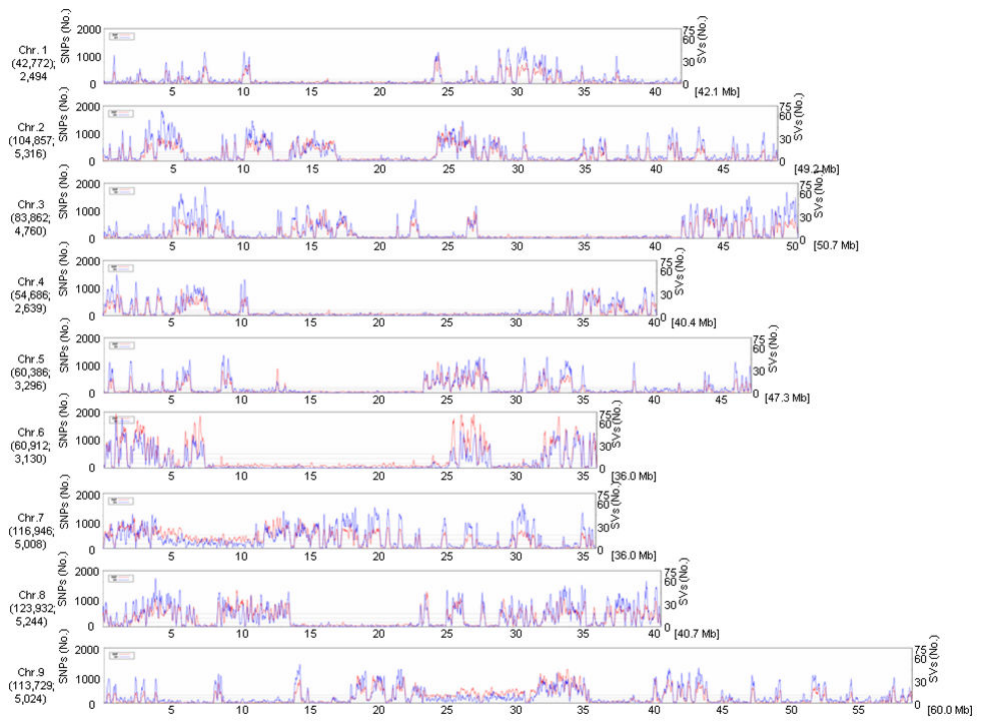
Distribution of SNPs and SVs detected between SLX and Yugu1 on the nine chromosomes. The *x*-axis represents the physical distance along each chromosome, split into 100kb windows. The total size of each chromosome is shown in brackets. The *y*-axis indicates the number of SNPs (left, red lines) and SVs (right, blue lines, InDels included). The total SNP and SV numbers in each chromosome are shown in parentheses.

The average densities of detected polymorphisms between SLX and the two reference genomes were 1899.0 SNPs per Mb and 2287.8 SNPs per Mb (SNPs), 66.8 Indels per Mb and 71.3 Indels per Mb (InDels), 25.2 SVs per Mb and 32.4 SVs per Mb (SVs) in the SLX genome, respectively (Table 2). The number of SNPs per Mb varied across individual chromosomes. Taking the SNPs detected between SLX and Yugu1 for an example, chromosome 7 had the highest density of SNPs, 3251.7 SNPs per Mb, and chromosome 1 had the lowest SNP density, 1014.9 SNPs per Mb (Table 2). Figure 3 shows the chromosomal distribution of the polymorphisms (SNPs, InDels and SVs) per 0.1 Mb. The distribution of polymorphisms was uneven within chromosomes. 268 high-density regions with >500 SNPs per Mb and 1 low-density region with <10 SNPs per Mb were identified. All chromosomes were composed of a mixture of dense and sparse SNP regions (Figure 3). For example, on chromosome 7, SNPs were dense from the region of 12.6 to 13.1 Mb (3,886 SNPs) and from 17.4 to 17.9 Mb (3,066 SNPs), but sparse from 23.2 to 23.7 Mb (37 SNPs) and from 24.1 to 24.6 Mb (45 SNPs). The distribution patterns of InDels and SVs densities were similar to those of SNPs (Figure 3), and the distribution patterns of three polymorphism types between SLX and Zhang gu were also similar to those between SLX and Yugu1 (Table 2 and Figure S1).

### Characteristics of SNPs, InDels and SVs

The SNPs detected between SLX and two reference genomes were classified as transitions (C/T and G/A) or transversions (C/G, T/A, A/C and G/T) based on nucleotide substitutions (Table 3). Both of the proportions of transitions (Ts) were significantly higher than the proportions of transversions (Tv). Among the Ts, G/A was slightly more than C/T, while among the Tv, both A/C and G/T were the most and T/A was the least. The Ts/Tv ratio was 2.6 and 2.5 between SLX and two references, Yugu1 and Zhang gu, respectively.

**Table 3:** Classification of nucleotide substitutions in the SNPs detected in SLX genome.

**Substitutions**	**SLX vs Yugu1**	**SLX vs Zhang gu**
Transitions(Ts)		
C/T	274,004	326,850
G/A	275,427	328,049
Tranversions(Tv)		
C/G	53,493	64,454
T/A	45,776	56,686
A/C	56,996	69,612
G/T	56,386	69,783
Ts/Tv ratio	2.6	2.5

**Figure 4:**
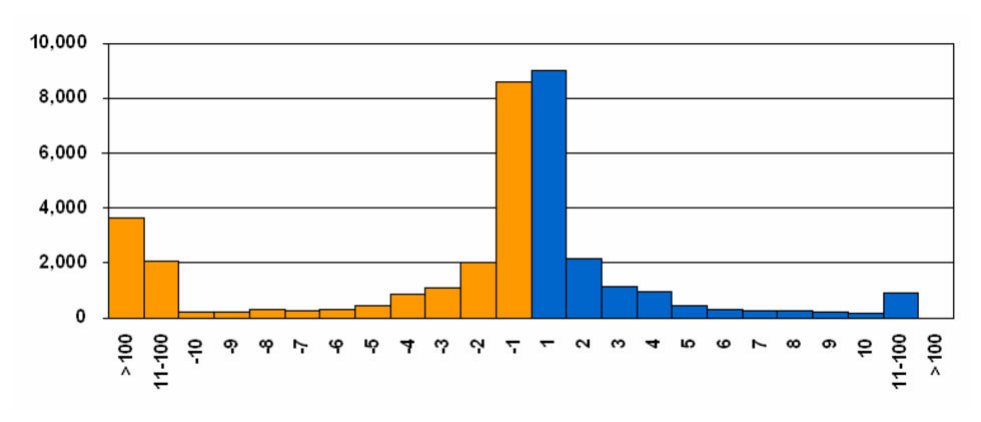
Distribution of the length of insertions and deletions polymorphisms identified between SLX and Yugu1 genome. The *x*-axis shows the number of nucleotides of deletions (orange) and insertions (blue). The *y*-axis shows the number of deletions or insertions at each length.

Among the 36,911 and 41,514 SVs (InDels included) detected in SLX compared with Yugu1 and Zhang gu, the percentage of the insertion (INS) and deletion (DEL) polymorphisms was 97.6% and 96.4%, respectively. The other types of SVs were interchromosomal translocation (CTX, 1.12% and 2.15%), insertion in the deletion (IDE, 0.77% and 0.85%), intrachromosomal translocation (ITX, 0.40% and 0.38%) and inversion (INV, 0.13% and 0.23%) (Table 4). Among the INS and DEL variations between SLX and Yugu1, the length of INS ranged from 1- to 188-bp, while that of DEL was up to 219,312 bp (Figure 4). Nearly a half of the INS and DEL polymorphisms (49.0%) were mononucleotide (INS—9,032 and DEL—8,616), 25.4% were 2- to 5-bp nucleotides (INS—4,702 and DEL—4,452), 7.3% were 6- to 10-bp nucleotides (INS—1,312 and DEL—1,331), 8.2% were 11- to 100-bp nucleotides (INS—899 and DEL-2,052) and 10% were >100-bp nucleotides (INS—8 and DEL—3,610). The INS and DEL detected between SLX and Zhang gu also showed the same trend as that between SLX and Yugu1 (Figure S2 and Table S1).

**Table 4:** Summary of SVs identified in SLX aligned with Yugu1 and Zhang gu reference genomes.

**Sample**	**SV**	**INS (%**)**^a^**	**DEL (%**)**^b^**	**CTX (%**)**^c^**	**IDE (%**)**^d^**	**ITX (%**)**^e^**	**INV (%**)**^f^**
**SLX_Yugu1**	37232	43.42	54.16	1.12	0.77	0.4	0.13
**SLX_Zhang gu**	41514	41.11	55.28	2.15	0.85	0.38	0.23

a, insertion. b, deletion. c, interchromosomal translocation. d, insertion in the deletion. e, intrachromosomal translocation. f, inversion.

### Annotation and effect of SNPs, InDels and SVs

**Figure 5:**
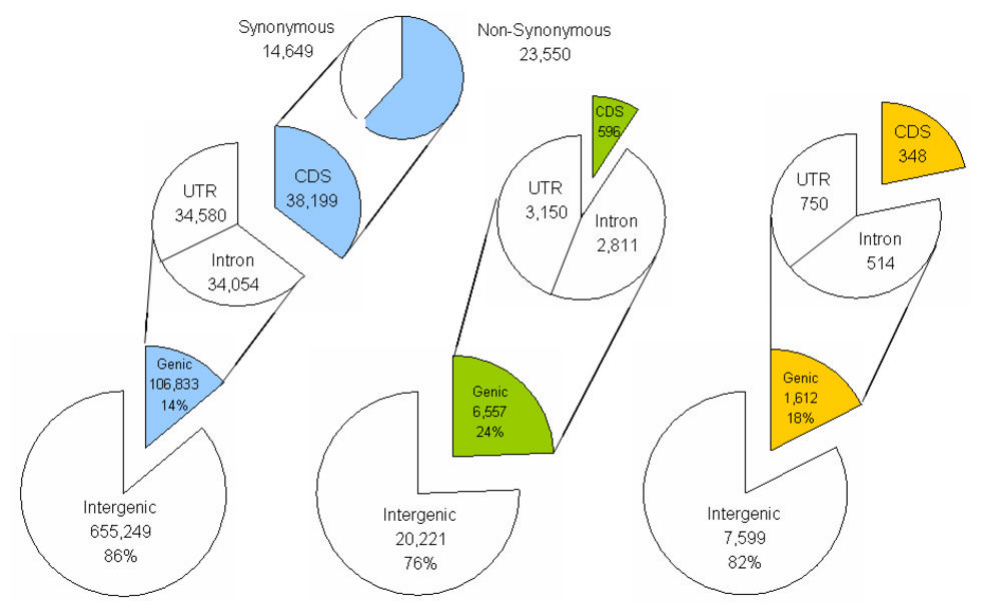
Annotation of SNPs, InDels and SVs identified between SLX and Yugu1. SNPs, InDels and SVs were classified as genetic and intergenic, and locations within the gene models were annotated based on the annotations of Yugu1 reference genome. The numbers and some proportions of three polymorphism types in each class are shown.

The use of the annotated reference sequences of Yugu1 and Zhang gu enable us to annotate the SNPs, InDels and SVs detected here, and to assign them with corresponding genes [34]. Among the polymorphisms between SLX and Yugu1, although 106,833 SNPs (14.0% of the total), 6,557 InDels (24.5% of the total) and 1,612 SVs (17.5% of the total) were located in gene regions, only 38,199 SNPs, 596 InDels and 348 SVs were found in coding sequences (Figure 5). However, among the polymorphisms between SLX and Zhang gu, 161,244 SNPs (17.6% of the total), 6,587 InDels (23.0% of the total) and 2,163 SVs (18.9% of the total) occurred in gene regions, but 57,265 SNPs, 521 InDels and 716 SVs were located in coding sequences (Figure S3). Totally, among the above 38,199 and 57,265 SNPs between SLX and two reference genomes, 23,550 and 35,313 SNPs were non-synonymous, which were located in 8,015 and 10,567 genes, respectively.

**Figure 6:**
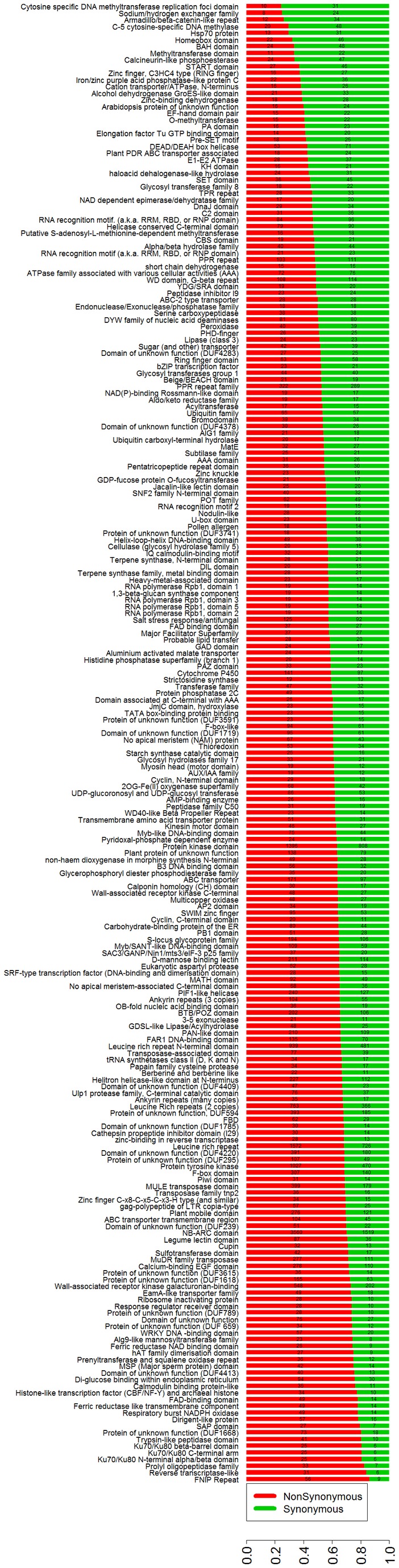
Annotation and distribution of non-synonymous and synonymous SNPs in SLX genome compared with Yugu1. The Pfam domains of foxtail millet genes with 30 or more nonsynonymous and synonymous SNPs were analyzed and listed. *x* ^2^ significance of the observed non-synonymous and synonymous SNPs for each Pfam group is *p-value* < 0.001. The Pfam genes are arranged according to the ratios of nonsynonymous to synonymous SNPs. The numbers in the red and green bars show the absolute numbers of the non-synonymous and synonymous SNPs in each Pfam domain, respectively.

SNPs are small differences but with great impact on the variation of genomes and the biological traits [22]. We therefore paid attention to SNPs in genic regions between SLX and Yugu1 and used the Yugu1 genome as a reference. Though the ratio of nonsynonymous-to-synonymous SNPs (ns/s SNPs) was 1.6 across all gene models, the ratio rose to 1.8 in Pfam-containing genes, indicating that the Pfam domains possibly had more amino acid substitutions (Figure 6). However, an opposite drop of the ratio was reported for rice and sorghum [14,22]. We further analyzed the distribution of the SNPs in Pfam-containing genes in detail. Figure 6 shows the number of non-synonymous and synonymous SNPs in each Pfam gene family. About 40% SNPs were found in NB-ARC domain (PF00931), protein kinase (PF00069, PF07714), and leucine-rich repeats (PF13855, PF08263 and PF12799) (Table S2). Among them, the sequences encoding NB-ARC domain have the most SNPs, and all the sequences encoding the above domains had higher ratios of ns/s SNPs than average, which was consistent with findings in *Arabidopsis* , rice and sorghum, indicating the diversification of plant disease resistance proteins is probably caused by pathogen pressure [14,17,22,36]. In addition, the sequences encoding Cytosine specific DNA methyltransferase replication foci domain (PF12047), Sodium/hydrogen exchanger family (PF00999) and Armadillo/beta-catenin-like repeat (PF00514) had the lowest ns/s SNPs ratios, and FNIP Repeat (PF05725) and Reverse transcriptase-like (PF13456) had the highest ratios.

**Figure 7:**
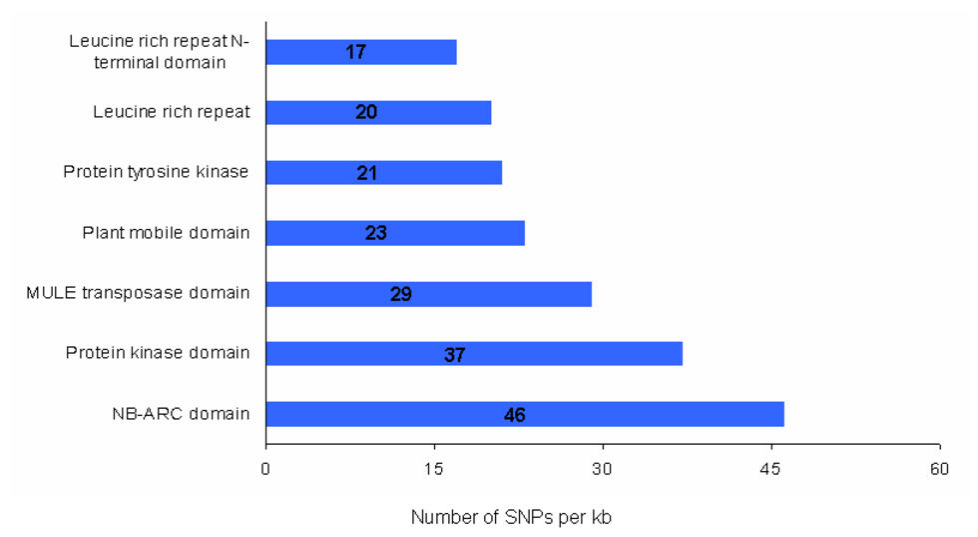
Annotation of large-effect SNPs in SLX genome compared with Yugu1. The numbers in the bars show the absolute numbers of large-effect SNPs for selected groups of genes. All the gene families shown were significantly abundant in large-effect SNPs (*p-value* < 0.001).

We also identified large-effect SNPs that were predicted to have a potentially disabling effect on gene function [17,22]: 456 SNPs were expected to induce premature stop codons, 25 to alter initiation methionine residues, 481 to disrupt splicing donor or acceptor sites and 96 to remove annotated stop codons, resulting in longer open reading frames [17]. Totally 2.4% of the annotated genes contained these large-effect SNPs [14] and the majority of the large-effect SNPs were enriched in NB-ARC domain (PF00931), protein kinase (PF00069, PF07714), leucine-rich repeats (PF13855, PF08263 and PF12799), transposase genes (PF10551, PF03108) and plant mobile domain (PF10536) (Figure 7), most of which were similar to the above results and consistent with findings in many plants [14,17,22,36–38]. This finding suggested that genes expected to adapt to survival environment might be more active in sequence variation than those essential for living in plant evolution.

**Figure 8:**
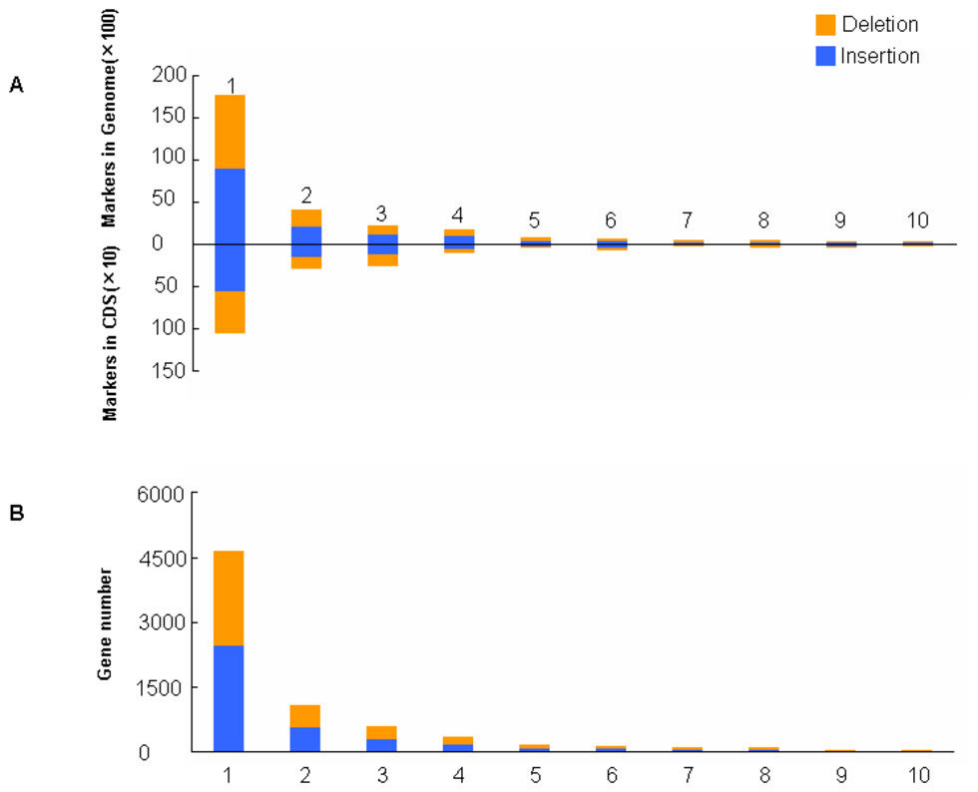
Distribution of IDPs in the SLX genome aligned with Yugu1 reference genome. A, Numbers of IDPs with different sizes in the whole genome and the CDS regions. B, Number of genes that contain IDPs with different sizes.

In addition to the above SNPs, SVs can also cause some large effects on genes in SLX. When increasing or decreasing nucleotides in a coding sequence, it’s possible to produce frame-shift mutation followed by character variation. Taking all the INS and DEL variations with 1- to 10-bp length, namely indel polymorphisms (IDPs), for an example, we examined their occurrence frequency in genomes and CDS regions [17]. As Figures 8A and S4A shown, with increasing IDP size, the number of IDPs decreased in SLX genome, despite of using Yugu1 or Zhang gu as references, which suggests that the number of IDPs preserved during evolution decreased with increasing IDP size. Zheng et al. [22] found that IDPs that are not multiples of 3 bp so as to produce frame-shift mutations are particularly uncommon in coding regions but relatively common in non-coding regions in sorghum. Here, we also found the trends in SLX compared with both Yugu1 and Zhang gu, especially in the latter comparison (Figures 8A and S4A), but both trends were less obvious than the reported in sorghum [22]. It suggested that IDPs with multiples of 3 bp were easier to be preserved in foxtail millet evolution because of their subtle impact on genes. In addition, with increasing IDPs size, the number of genes containing corresponding IDPs decreased (Figures 8B and S4B), not consistent with the reported results in sorghum [22], which might be attributed to the imperfection of the foxtail millet genome annotations.

### Validation of polymorphisms and development of new DNA markers

The SNPs identified in SLX compared with Yugu1 were expected to be useful in genome-wide genetic analysis. To verify the accuracy of these SNPs, 50-70 SNPs were randomly selected in each chromosome, resulting in a total of 465 SNPs, covering both intergenic and genic (UTR, intron and exon included) regions, and including both non-synonymous and synonymous SNPs (Table S3). We designed primers according to the flanking sequences of selected SNPs and amplified the target sequences by PCR, using SLX genome DNA as template. The specific target bands were successfully amplified corresponding to each pair of primer. After sequencing each specific band, we found that 462 SNPs showed expected nucleotide substitutions, that means 99.35% examined SNPs being verified (Table 5), indicating that the quality of the SLX re-sequencing data was reliable.

**Table 5:** The validation result of 465 SNPs identified in SLX compared with Yugu1 genome.

**Chromosome**	**The number of detected SNPs**	**Verifying result**	
		**True SNP**	**False SNP**
chr01	54	54	0
chr02	54	54	0
chr03	42	42	0
chr04	59	59	0
chr05	45	45	0
chr06	66	65	1
chr07	50	50	0
chr08	43	43	0
chr09	52	50	2
Total	465	462	3

In addition, we did a *de novo* assembly with high-throughput sequencing reads, and then validated structure variations such as inversions detected between SLX and Yugu1. The final assembly contained 96,778 contigs with more than 500 bp that cover 161 Mb of the genome with N50 of 2.3 kb (Table S4). About 70% inversions were confirmed being existed in the genome with both plus and reverse contig-matches (Table S5), and the rest may be due to the low-depth sequence coverage or assembly errors, which indicates that most of the structure variations detected by both pindel [32] and BreakdancerMax.pl [33] softwares in the way of the re-sequencing analysis were accurate and applicable.

**Figure 9:**
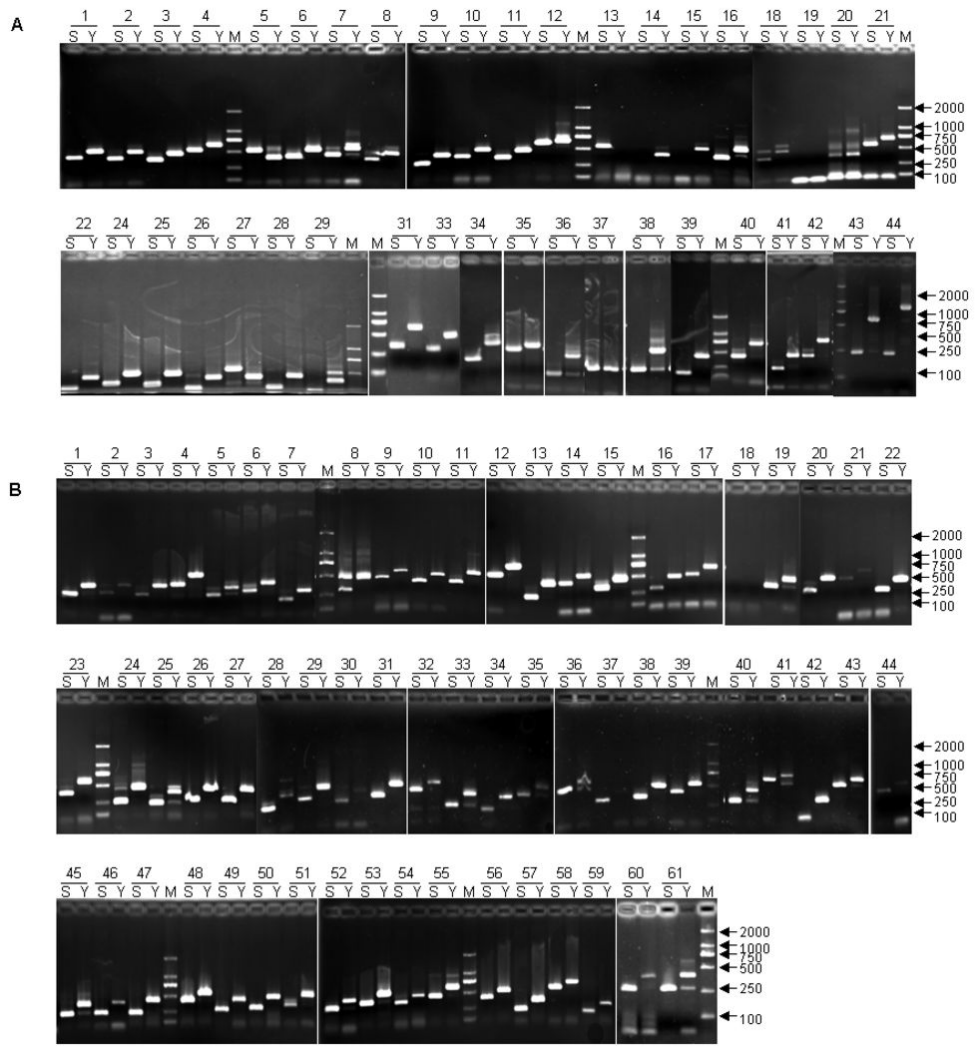
Validation of SV polymorphisms in SLX and Yugu1 genomes. A, The electrophoresis gel image of PCR amplification of 29 newly developed SVs distributed on the 4th chromosome. B, The electrophoresis gel image of 61 newly developed SVs distributed on the 7th chromosome. Based on SVs obtained by SLX compared with Yugu1, we selected 29 (A) and 61 (B) INS and DEL variations with 100- to 200-bp in length to develop new DNA markers. S represents SLX, Y represents Yugu1, M represents DL2000 molecular makers.

**Figure 10:**
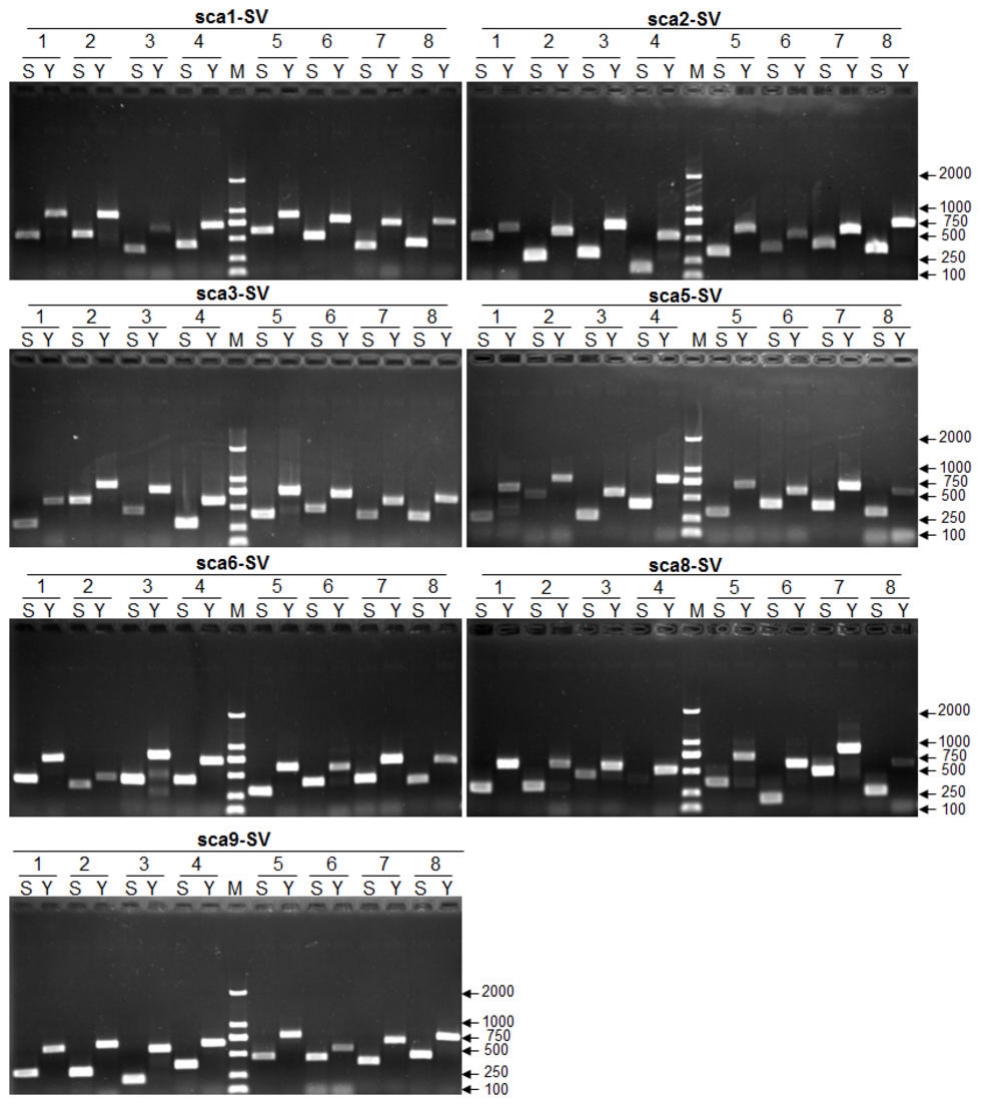
The electrophoresis gel image of PCR amplification of 56 newly developed SVs markers in SLX and Yugu1 genomes. Based on SVs identified between SLX and Yugu1, we selected 8 DEL variations with 100- to 400-bp in length from each chromosome (1st to 9th excluding 4th and 7th) to develop new DNA markers. S represents SLX, Y represents Yugu1, M represents DL2000 molecular makers.

Furthermore, based on SVs obtained by SLX compared with the two references, we screened and listed the INS and DEL variations with 100- to 400-bp in length, which were suitable and convenient to be used for molecular markers (Tables S6 and S7). Next, we first selected 29 and 61 INS and DEL variations with 100- to 200-bp in length from the 4th and 7th chromosomes, respectively, to develop some convenient markers for validation and further application (Table S8). Primer design and PCR amplification were followed as the above SNPs detection. As Figure 9A and 9B show, more than 92% primers could amplify the target product in both SLX and Yugu1 genome DNA templates, and positive rate of the expected polymorphism was 92%, further indicative of the reliable quality of the SLX re-sequencing data. Furthermore, in order to make better use of the SV markers, we also developed 8 DEL variations with 100- to 400-bp in length from each of the remaining chromosomes (Table S9). As Figure 10 shows, all primers could amplify the single target product and all the SV markers could show clear length polymorphism in both SLX and Yugu1.

### Fine-mapping of a waxy gene in SLX using the new developed DNA markers

**Figure 11:**
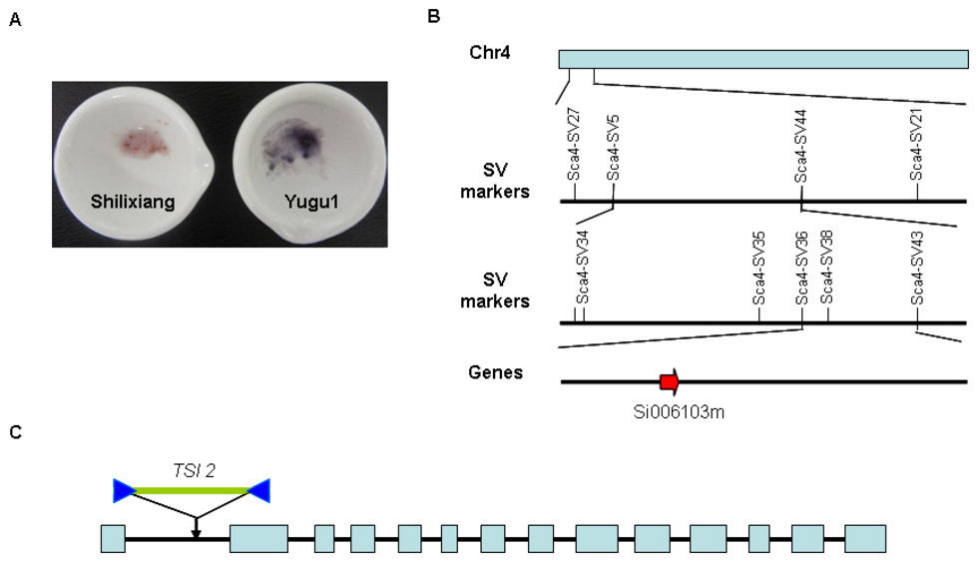
Map-based cloning of *waxy-slx*. A, Waxy trait of SLX compared with the non-waxy type of Yugu1 by staining their seeds in the I_2_/KI solution. B, A fine genetic map of the *waxy-slx* region on chromosome 4 by new developed SV markers. C, A *TSI2* transposable element insertion was found in the first intron of the *starch* *synthase* gene (Si006103m).

Waxy-type cereals are characterized by little or no amylose, which constitute about 20% or more of the total starch in the non-waxy endosperm. SLX is a waxy landrace in which amylose content of endosperm is only 1.22% [27], while Yugu1 is a non-waxy cultivar as shown by staining their seeds in the I_2_/KI solution (Figure 11A). In previous studies, we constructed the F_2_ population, with size of 460, of SLX and Yugu1 to map the waxy locus in SLX. In the 460 F_2_ populations of SLX and Yugu1, the segregation of non-waxy trait compared with waxy trait was about 3:1 by I_2_/KI staining (Table S10), which suggests that waxy trait is probably controlled by a single recessive nuclear gene, namely *waxy-slx*. The gene was then mapped onto the 4th chromosome (data not shown).

Availability of a genome sequence could facilitate gene mapping. We used 29 newly developed SV markers to further narrow down the waxy gene in SLX to a 3’-flanking region of the sca4-SV5 marker (Figure 11B). For fine-mapping, we chose 6 additional SV markers (Table S8) located in the 3’-flanking region of the sca4-SV5 marker, and genotyped them in the F_2_ population. The waxy gene was located between sca4-SV36 and sca4-SV43 (Figure 11B), near the sca4-SV38 marker, within a ~145-kb region. Twenty-three predicted genes were located in this region, including Si006103m, which was annotated as *starch synthase* encoding granule-bound starch synthase 1 (GBSS 1). We amplified the sequence of Si006103m in SLX by PCR, and found that a *TSI-2* transposon with 5258 bp was inserted into the first intron of Si006103m and the rest nucleotides in this gene were almost identical to those in the Yugu1 (Figure 11C and Figure S5), which indicates that there is a iv type waxy gene in SLX [39]. Therefore, by mapping a waxy gene in SLX using our newly developed markers, we have successfully shown that the re-sequencing data can be used to accurately identify genes we are interested in.

### Potential applications of sequence data in foxtail millet breeding

The world population is growing faster than ever, therefore, crop breeding is particularly important for improving yield and adapting to various agro-climatic conditions to ensure the food security of the world [23,40]. However, the traditional breeding method, currently the major breeding approach, is time-consuming and labor-intensive. For example, it took us nearly 15 years to stably transform both rust-resistant and waxy traits from SLX into an elite cultivar, Yugu1, through multiple-crossing breeding method [Yugu1× (sterile line1066A×SLX) F_4_] [27]. Traditional breeding process can be greatly accelerated by marker-assisted selection (MAS) breeding. However, there were few molecular markers available for foxtail millet before the completion of the two reference genomic sequences of foxtail millet. Therefore, both of the research on mapping and isolating agronomically important genes and MAS breeding in foxtail millet lagged behind that of the other crops, such as maize and rice.

The completion of the reference genomic sequences of important crops, followed by high-throughput re-sequencing of their diverse germ plasm resources, has accelerated the process of crop improvement. The recent release of the two reference genomic sequences of foxtail millet, Yugu1 and Zhang gu [10,11], followed by re-sequencing its wild and cultivated varieties, will also benefit the improvement of foxtail millet. In the present study, we conducted re-sequencing and genome-wide variation analysis of a foxtail millet cultivar, SLX, to investigate the genetic structure, develop new molecular markers and explore the agronomic traits-related genes in SLX for foxtail millet improvement. The molecular markers (SNPs, InDels and SVs) we have identified in this study should be invaluable for both breeding and identifying agronomically important genes in SLX and other related foxtail millet.

In fact, most agronomically important traits are determined by multiple loci (each with a relatively small effect), named quantitative trait loci (QTL) [41]. It is important to rapidly identify each locus or major locus of QTL for efficient crop breeding by MAS, which is made available by the rapid development of high-throughput sequencing-based genotyping technologies on the whole-genome scale [23,24]. Currently, there are many methods used in QTL identification, including genome-wide association studies (GWAS) [25,42], QTL-seq [43], MutMap [23], SHOREmap [15], NGM [44], and others [45,46]. With the further rapid development in sequencing technology, the existing and emerging QTL identification method will be attractive for marker/genomics-assisted crop breeding in a quick and cost-effective manner, which will speed up crop genetic improvements, including foxtail millet of course.

## Materials and Methods

### Sample preparation

Shi-Li-Xiang (SLX), a landrace cultivar of foxtail millet [ *Setaria* *italica* (L.) Beauv.], was used in this study. A hybrid population of 460 individuals was bred from SLX and another cultivar, Yugu1. The F_2_ population was used for mapping analysis.

### DNA isolation and genome sequencing

Genomic DNA was extracted from young leaves of SLX and Yugu1 using a modified CTAB method [47]. The DNA from SLX was then randomly sheared. After electrophoresis, DNA fragments of the desired length were gel-purified. Adaptor ligation and DNA cluster preparation were performed and subjected to the Solexa sequencing using Illumina Genome Analyzer ii [48]. Low-quality reads (<20), reads with adaptor sequence and duplicated reads were filtered, and the remaining high-quality data were used in the mapping.

### Mapping of reads to the reference

The pair-end (PE) sequencing reads were aligned to the Yugu1 and Zhang gu reference genome sequence separately using BWA software algorithm under the default parameters with a small modification: allowing no more than three mismatches totally in the sequence and not allowing gap (-o 0) [30]. Reads that aligned to more than one position of the reference genome were filtered and used for determining reads mapping to multiple positions in the reference and unmapped reads [35]. Average sequencing depth and coverage were calculated using the alignment results [48]. The mapped reads were then used to detect SNPs, InDels and SVs polymorphisms.

### Detection of SNPs, InDels and SVs polymorphisms

Firstly, we used SAMtools software [31] to detect SNPs with the following parameters: samtools} mpileup -6 -f reference. fa -D -C 50 -g -s -u bcftools view -b -c -e -g -I -v. The above detected SNPs were then screened under the following criteria: no less than 2× for coverage depth (no less than 3× in the heterozygous locus), no more than three times of average depth (11×), and discarding the SNPs detected in the repeat region.

Secondly, SVs were detected using both pindel [32] and BreakdancerMax.pl [33] softwares with their default parameters. For obtaining reliable SVs, the detected SVs must be returned to the PE alignments between SLX and the reference, and be validated under the following criteria: 2× to 100× for coverage depth and more than 20 for SVs Quality. In our result, the types of SVs include insertion (INS), deletion (DEL), interchromosomal translocation (CTX), deletion including insertion (IDE), intrachromosomal translocation (ITX) and inversion (INV). And in our analysis, InDels were defined as the insertion or deletion the length of which was from 1 to 5 bp.

### Annotation of SNPs, InDels and SVs

The localization of SNPs, InDels and SVs were based on the annotation of gene models provided by the two reference genome databases [10,11]. The three types of polymorphisms in the gene region and other genome regions were annotated as genic and intergenic, respectively. The genic SNPs, InDels and SVs were classified as CDS (coding sequences), UTR (untranslated regions) and introns according to their localization. SNPs in the CDS were further separated into synonymous and non-synonymous amino substitution using Genewise version [49]. The GO/PFAM annotation data were further used to functionally annotate each gene including non-synonymous SNPs or SVs with 1- to 10-bp lengths [10,11].

### Validation of SNPs and SVs

50-70 SNPs were randomly selected in each chromosome and verified with SLX genome by sequencing. Firstly, the flanking sequences of the selected SNPs (one or many) were ensured to be unique. Second, primers were designed to amplify the sequences containing examined SNPs. Next, target sequences were amplified by PCR using SLX genome DNA as template, and then amplified specific products were recovered, purified and sequenced. Finally, SNP sites were analyzed based on the sequencing data. All of the INS and DEL variations with 100- to 200-bp in length from the fourth and the seventh chromosomes and 8 DEL variations with 100- to 400-bp in length from each of the remaining chromosomes were selected. Primer design, PCR amplification with both SLX and Yugu1 genome DNA as templates and electrophoresis detection were followed as the above SNPs detection.

### Genome assembly and inversions validation

First, a *de novo* assembly with high-throughput sequencing reads of SLX was carried out. Using ABySS assembler [50], the *de Bruijn* graphs were constructed for all k-mer sizes between 30 and 70 with 5-bp intervals. The contigs generated from 30 k-mer were chosen to do the alignment analysis. Due to the low depth genome coverage of SLX sequencing data, the final assembly contained 96,778 contigs with more than 500 bp that cover 161 Mb of the genome with N50 of 2.3 kb.

Secondly, the validation of the inversions was performed in the following way. The inversion sequences were extracted from Yugu1 genome according to our results and sequence similarity searching was performed against the contigs using standalone BLAST from NCBI. Next, the high-scoring segment pairs (HSP) from blast output which e-value larger than 1e-20 or identity smaller than 98% or match length smaller than 200 bp were discarded. Finally, the plus and reverse strand coverage ratios against inversion sequences were calculated.

### Trait mapping of waxy trait

We used the F_2_ population (460 individuals) obtained by crossing between the SLX (waxy trait) and Yugu1 (non-waxy trait) strains. Waxy phenotype data were collected in the F_2_ population detected by staining their seeds in the I_2_/KI solution. Fifteen seeds of F_2_ individual were separately ground with pestle in the mortar. If each endosperm of seeds was dyed pink by I_2_/KI solution, the phenotype of the F2 individual was considered as waxy. If all of them were dyed dark blue, the phenotype of the F_2_ individual was considered as non-waxy. If their colors were segregated in terms of staining by I_2_/KI solution, the phenotype of the F_2_ individual was considered as heterozygous.

### Accession number

Raw sequence data obtained in our study have been deposited in the NCBI Short Read Archive with accession number SRA072113. Sequences of *waxy-slx* (Si006103m) in SLX have been sent to NCBI with accession number KF372879.

### Note

Recently, Jia et al. [51] reported low-pass re-sequencing of 916 diverse foxtail millet varieties with 0.7× coverage on average. They identified 0.8 million common SNPs, constructed a haplotype map of the foxtail millet genome and performed genome-wide association studies on 47 agronomic traits. SLX was one of the 916 foxtail millet accessions with 0.7× coverage re-sequencing data. Our work provided about 11× coverage data.

## Supporting Information

Figure S1 **Distribution of SNPs and SVs (InDels and SVs) detected between SLX and Zhang gu on the nine chromosomes.** The *x*-axis represents the physical distance along each chromosome, splitting into 100kb windows. The total size of each chromosome is shown in brackets. The *y*-axis indicates the number of SNPs (left, red lines) and SVs (right, blue lines, InDels included). The total SNP and SV numbers in each chromosome are shown in parentheses.
(TIF)

Figure S2 **Distribution of the length of insertions and deletions (INS and DEL) polymorphisms identified between SLX and Zhang gu genome.** The *x*-axis shows the number of nucleotides of DEL (orange) and INS (blue). The *y*-axis shows the number of DEL or INS at each length.
(TIF)

Figure S3 **Annotation of SNPs, InDels and SVs identified between SLX and Zhang gu.** SNPs, InDels and SVs were classified as genetic and intergenic, and locations within the gene models were annotated based on the annotations of Zhang gu reference genome. The numbers and some proportions of three polymorphism types in each class are shown.
(TIF)

Figure S4 **Distribution of IDPs in the SLX genome aligned with Zhang gu reference genome.** A, Numbers of IDPs with different sizes in the whole genome and the CDS regions. B, Number of genes that contain IDPs with different sizes.
(TIF)

Figure S5 **The sequence information for the *waxy-slx* locus.** There are 9467 bp nucleotide bases including 14 exons, 13 introns and a *TSI-2* transposon insertion. The gray-labeled nucleotides indicate exons. The unlabeled nucleotides indicate introns. The dark blue-labeled nucleotides indicate the *TSI-2* transposon, which is inserted in Intron 1. The underlined and bold nucleotides in red color, ‘ATG’ and ‘TGA’, indicate initiation codon and stop codon, respectively. The sequence has been sent to NCBI with accession number KF372879.
(DOC)

Table S1 **Distribution of the length of insertions-deletions (INS and DEL) polymorphisms between sample and two reference genomes.**
(XLS)

Table S2 **The List of the Pfam gene families with 30 or more nonsynonymous and synonymous SNPs in SLX genome compared with Yugu1.**
(XLS)

Table S3 **475 SNP markers between SLX and Yugu1 used to validate the accuracy of resequencing data for shilixiang.**
(XLS)

Table S4 **Summary of genome assembly.**
(XLS)

Table S5 **Validation of the inversions detected between SLX and Yugu1.**
(XLS)

Table S6 **The list of the INS and DEL variations with 100- to 400- bp in length identified between SLX and Yugu1.**
(XLS)

Table S7 **The list of the INS and DEL variations with 100- to 400- bp in length identified between SLX and Zhang gu.**
(XLS)

Table S8 **SV markers from chromosome 4 and 7 between SLX and Yugu1 used to validate the quality of re-sequencing data for SLX.**
(XLS)

Table S9 **SV markers from chromosome 1 to 9 (chromosome 4 and 7 excluded) between SLX and Yugu1 developed for application.**
(XLS)

Table S10 **Phenotype of waxy trait and genotypes of nine sca4-SV markers in the F2 populations.**
(XLS)
