# Supplementary figures and images for: Identifying the Genome-Wide Sequence Variations and Developing New Molecular Markers for Genetics Research by Re-Sequencing a Landrace Cultivar of Foxtail Millet
Source: PLoS One. 2013 Sep 10;8(9):e73514. doi: 10.1371/journal.pone.0073514 (PMC3769310; doi:10.1371/journal.pone.0073514)

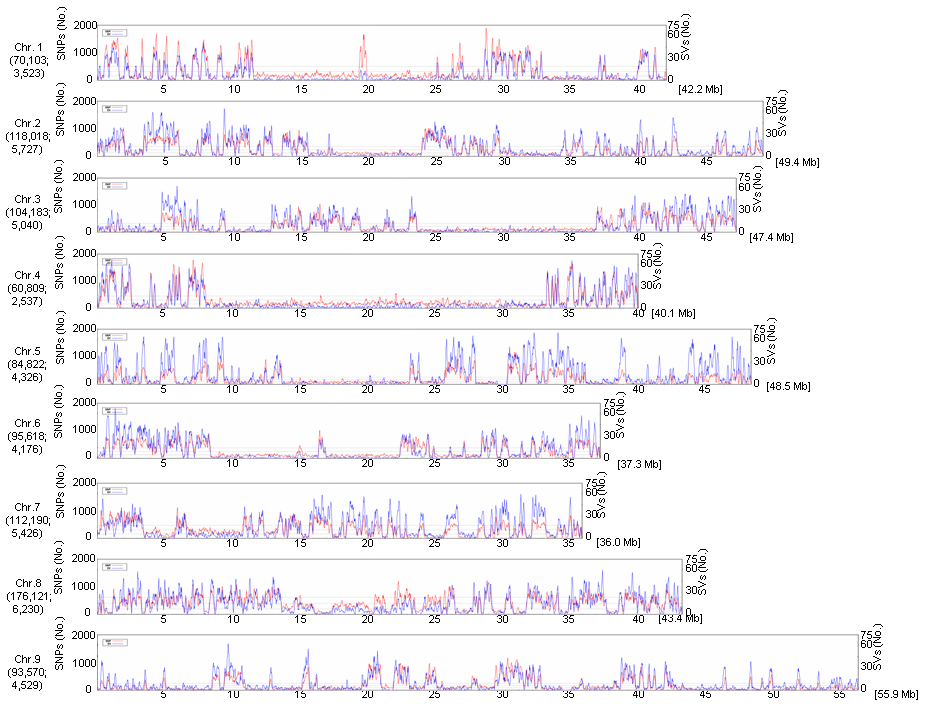

Supplement: Figure S1 — Distribution of SNPs and SVs (InDels and SVs) detected between SLX and Zhang gu on the nine chromosomes. The x-axis represents the physical distance along each chromosome, splitting into 100kb windows. The total size of each chromosome is shown in brackets. The y-axis indicates the number of SNPs (left, red lines) and SVs (right, blue lines, InDels included). The total SNP and SV numbers in each chromosome are shown in parentheses. (TIF) [file pone.0073514.s001.tif]

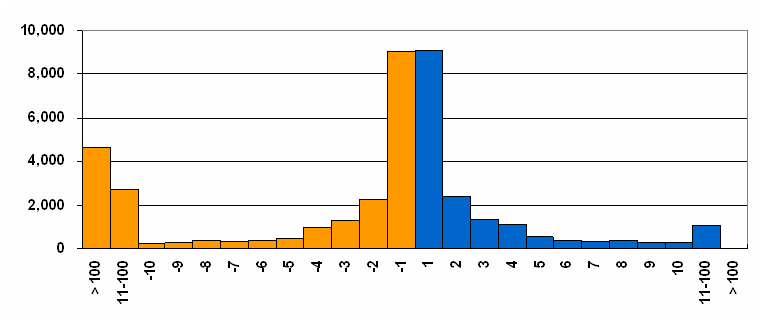

Supplement: Figure S2 — Distribution of the length of insertions and deletions (INS and DEL) polymorphisms identified between SLX and Zhang gu genome. The x-axis shows the number of nucleotides of DEL (orange) and INS (blue). The y-axis shows the number of DEL or INS at each length. (TIF) [file pone.0073514.s002.tif]

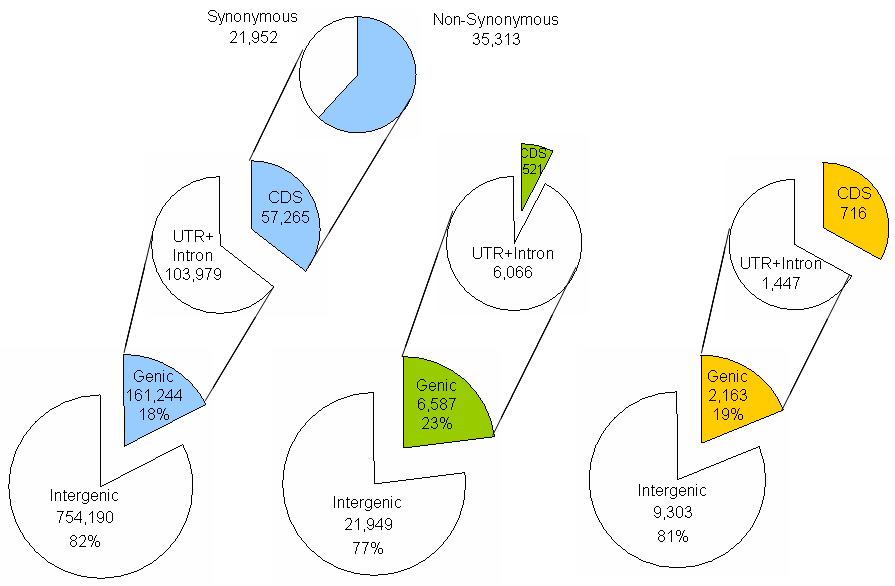

Supplement: Figure S3 — Annotation of SNPs, InDels and SVs identified between SLX and Zhang gu. SNPs, InDels and SVs were classified as genetic and intergenic, and locations within the gene models were annotated based on the annotations of Zhang gu reference genome. The numbers and some proportions of three polymorphism types in each class are shown. (TIF) [file pone.0073514.s003.tif]

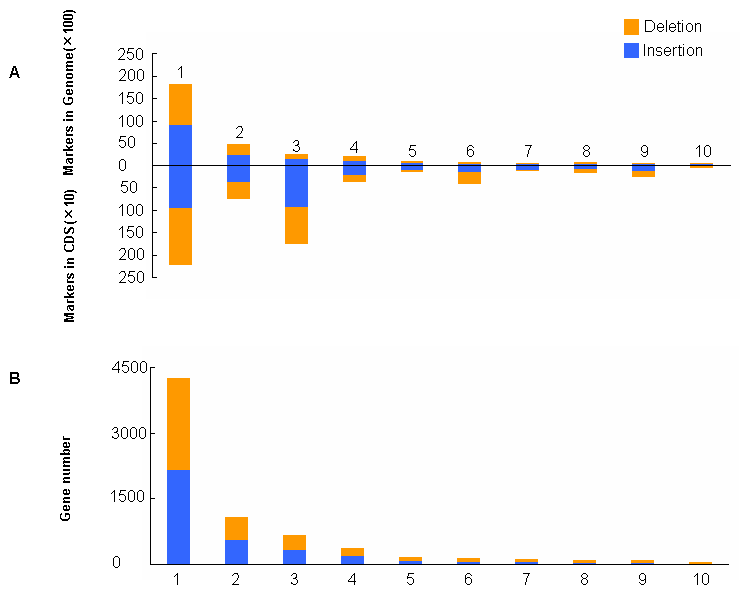

Supplement: Figure S4 — Distribution of IDPs in the SLX genome aligned with Zhang gu reference genome. A, Numbers of IDPs with different sizes in the whole genome and the CDS regions. B, Number of genes that contain IDPs with different sizes. (TIF) [file pone.0073514.s004.tif]
