# Supplementary material for: Identifying the Genome-Wide Sequence Variations and Developing New Molecular Markers for Genetics Research by Re-Sequencing a Landrace Cultivar of Foxtail Millet
Source: PLoS One. 2013 Sep 10;8(9):e73514. doi: 10.1371/journal.pone.0073514 (PMC3769310; doi:10.1371/journal.pone.0073514)
Supplement: Figure S5 — The sequence information for the waxy-slx locus. There are 9467 bp nucleotide bases including 14 exons, 13 introns and a TSI-2 transposon insertion. The gray-labeled nucleotides indicate exons. The unlabeled nucleotides indicate introns. The dark blue-labeled nucleotides indicate the TSI-2 transposon, which is inserted in Intron 1. The underlined and bold nucleotides in red color, ‘ATG’ and ‘TGA’, indicate initiation codon and stop codon, respectively. The sequence has been sent to NCBI with accession number KF372879. (DOC) [file pone.0073514.s005.doc]

> *waxy-slx*

1 CTCCACACCA CCACCAAGGG TTTGAAGAAG AGGAAGAACT AAACCACTGC AAGCCAGTGA

61 CGGATCGACG ACGACACGCC CACCCCGGGC TCCCTGCTCA TCCATCCGTC AGGTACGCCG

121 CACTCCCTGC TCGACCGAAT CCTGGGCTTT CATTTCCCGT ACCAGTTCCT GAATTCAGAC

181 ATGTATTTGA GTTGTTCATG TGGTAAATGT CTAGAGATTG GGTCATCAGA TCCTTAACTT

241 GTGTGGTCGT ACATATTTCC GTTTCGGTCG GTAGGAAGTG CAGGTGCATG CCTCTAGTTT

301 ACATCAAGTT TTTTTTTTTC CTTTTTCGAT CCAGTTCGTT TGCCTGTTTC GTATTCTGTC

361 TGAAATCTGA GTCCATGCCG ATGTATAGGA AGTGGATGCC TTTAGTTTAG ATCAAGGTTT

421 CTAGATCCAG TTTGTTCGTG TGCTTCGTGT TCTGTCTGAA TCTAGCTAGG CCTCTGTTGG

481 AGTGGATGGG GGTGTTTGGG AGGGGGGTGC TAAAATTTAG CACCCCCATT TTAGTCACTT

541 TTAGCCTCTC CTCCTCCCAA ACACCTAGGC TAAAATGGAG GGGCTAAATT TTAGCCCCCC

601 CATAATCCAT TAGCCCTTCA AGAGGTGCTA AAATGGACTA CAAGTGCTAA AAGTGGTCCC

661 CCTCCTTCAT ATTTCCACCG GTACCCCCAC TCCCTTCCCC ATCCTGTCTC CTCCCACTCC

721 CGCACACGCG CCGCCACTCC CTTCCCTCCC GTCTGGATCT CGCCCCCTCC TCCGCTCCCT

781 CCGCCGTGCC CGATCCGCCG CTCCCTCCGG CGACGCGCCT CCGCCGCCCC TCTGCCGCTG

841 CGCCTCCGCC GCCCCTCCGC CGCGCCCTCC GCCTCACTCA CTCCTCCGCG CCCCTCCGCC

901 GCGCCTCCTC CTCCGCATCC ATCCGCAGCG CCCCCCCCTC GCCTCTCCCT CCGTCGCTCC

961 CTACGCCGGT TCCTCCGCCG CTTCCTACGC CGCTCCATCC GCCGACGCGA CTCCGCCGCC

1021 CCGCCGCCGC TCCCTCCGCC GCGCCCCCTT TGTCGCCCTC CTTTTCCTCC CCACCACGCC

1081 GCCTTCCTTT CGTCGGCTCG TCGCCACTCC GCCGCCGCTC CCTCTGCCGC GCCCCCTCAG

1141 CCGCGCCTCC TCCGCCGTCC GTCCCTCGCG CCCCACTCCA CGCCGCGCCC CACTCCGCCC

1201 CAGCTTCAGA TCTAGAAGCA GCGCGTCGTC ATGGACGGCA ACGGCGACGA CCACTTCTCG

1261 CAGCCGGATC CGGCCTTCCC CTCCGGCGCC TTCGACCTGT ACTCCCAGGC GGATTCGTAT

1321 CAAGGACCAC GATCCGGGAT GCAGGCCTTG GATCTGAACT CCCAGGTCGA CGAGTTCCCC

1381 GCCTTCAGCT CGTACGCCGA CATCCTCCGG AGTGACCACG GCGGAGTTGC TCGAGGGGGC

1441 GGTGCTCGCA CACCCGGGCT TCGCGTGGCC CACAATGGCG GCGGAGACAG CAGAGGAGGA

1501 GGCGCCACGA GAGGTGGCGG CGCAAGTGCT GACAAGGGTC GCGGCGCCGG GGGGAGCAGA

1561 GGATGCGGCG CCGCGAGAAG CAGAGGTGGT GGTGCAAACC GTGTCGGCGG CACTGGCGGT

1621 AGCAGAGGTC GTGGGCGGGC GACTAGGAAC CTATTCATTG GTTCCGCGTC TGCTGGTGGT

1681 GCTGCCGGCG GCGGTGGACG CGGCGGTGGA CGTGTGGGTG GCCGTCGTCG ACGTGGAGGC

1741 AGTGACCATG AAGATTATGT GTATGCTGAC GAAGGATACG CGCCAGAGGT TGCTGATGGC

1801 GTGGAAGTGA TATTTCCTGG CTCTTCAAAG GTAATTATCT AAACCTGAAG TCCTGGAATG

1861 TTGAATGTTG ATTCATTTGA AGCTCCGCTA CTGAACTTGA TGTTTGTGAT AATCTGAACA

1921 TTCGTATTCA GTAGTGTGAA TATCTGTTGA CTGATAATCA GATGTTTGTA TATGTCAGCT

1981 ACTCAATCCT CCCAAGTACT TTACACCTCT TAATATGAAA TGCTAGTGTG CTGGAAGACT

2041 TGCTTGGTTT GTTTACTATA CATATGGTTT CAACAATGAA TATGCTTCTT CGTTTTACTG

2101 GTCATATTAT TTGGGAAATA TGGTTACATG TGGTCAGGAT CCATACCTCA ATTGATATTG

2161 CTCTCCTCAA TTGTTGTTGA ACCTTGATGT ATGAATGTTT CCTGTACGAA TGTTGTTGTT

2221 GAACCTTGAT GTATGAATGT TTCCTGACTT TAGTATTTCT TTTTTAACAC AACTCCAGTA

2281 GGAACCTTGT TGTGGAATGC TGACCATAGG AAATTCTTAC TGACCATAGC TAGTTGACTG

2341 GTCATATTAT ATTGATCTGG TTGTATATGT GTATATGACT GTAGTTTGAG TACAAATTCT

2401 TTCTGACCAT AGCTAGAAAT TCTTACTGAC CATAGGTAGA AATTCTTACT GACCTGACTA

2461 TAGCTACAAA TTCTTACTGA ACATAGCATA CAACATACAT ATTGATAAAT GAAGTCCTTG

2521 TATGACTCTT TCATATCCTT GTGTGTTTGC ACTAGATTAC CTATGACAAG GCATATTGGT

2581 CAGAACCAAA TACACTTTTG TTCTGTAATT TGGTCGTTGA GCAAATTAGA GAAGGGAATT

2641 GTCACAAAGC TACTATGTCC GCAAGAGGTT ACAAGCTAGT GCAAGAAAAA TATTATGCTG

2701 CAACTGGATT GAGACATAAA AGGAGGCAGT TCAGGAATAG GATACATGGA TTGAAAACAT

2761 TGTACACCTT CTGGAGGGGC TTGCAGAAAG AAAGTGGCTT GGGAAAGGCT TCAAATGGTA

2821 CTGTCACAGC TACAGATACC TGGTGGGAGA ATAACACTAA GGTAAGCCAC AGCTTCAAAT

2881 GCTACTGACT GTGGTATAAA AACACAAAGG TAACCTGTCT TGCATTTGTT TTGCAGGGTC

2941 CCAAATCAGA ATGTAAGAAG TTCAGATATG GTGTTCCAGA GTACATGGAC CATCTTGAGG

3001 AGATATTCCA TGAGGTTGTA GTAGATGGGT CTACCTCCTT CATACCAGGA GAAGAAGATG

3061 AAGAACCTGA AGAAGAAGAT GAAGAACCTG AAGAAGAAGA TGAAGAGCTT CCTGGAGAGC

3121 AGGGGTTCCC TGCTGATTAT GATGACAGCC CTGGTAGTAC TAATAGCAGA AAGAGGCTTA

3181 GTAGCAACAG CACTAGGTCC ACTGCCTCAA GTCCTGGAAA GAAGTCCAAG AGTCCTATGG

3241 TTCAGGTTAT GGACAAGATG TTGAATAAAT GGGCTGAGTC TGATTCTAGG CACCAGAAGA

3301 TACTGAAGAA GAAGGTGGAT GTCAAAGCTA GCAAGGAGAT GCAGGAAAGA GCAGAGTTGA

3361 AGAAGTGTCA ACAATTGGCC ATAGAATGTG GTGCTGCAGC TGATAGTGTA GAGTACTATG

3421 CTTGCCTGAG CATTTTTAAA GATGGATTGC ACAGGGAGTT CTTCTGCAAT ATCCCAAGTC

3481 CTGAAGCTAG GCTGGTTTTC CTGAAGAGAT GGTGCGAGGA ACACAATATG TACTAGTTTA

3541 GGGCCTGTTG AACTAAGATG TTTGCTAGTT TAGGGCCTGT TGAAGAGACA GGTCTAAGAG

3601 ATGTTTGCTA GTGTGTTTGA AATGAGACTG ATTGCACTGA GTTGGTCCAA CTGAGTTGTT

3661 TGAACTGTGA ACCATGTTGT TTGAATCAAA TTAGTAGTTG GTTGAATTGA GTATCTGTTG

3721 AACTGTGAAC CATGTTGTTT GAATAAAATA AGTAGTTGGT TGAACTGAGT TGTTTGAACT

3781 GTGTCTGATA TGCAAACCTA AATGAGTACT AACCTTTTGC ATTGTTTATG ATTTAGGATG

3841 CTAGTTCTTC AACTGAGTTG TCAAGTGATG ATGAGGATAA TCATATTATG GAAGAATCAT

3901 CTAGTGAAGA TGAAACTATG GAGCTCATAT TGCACTGTCG TAAGAGGAAT CGGGAGTTCG

3961 TCCAAATGAT GTTAACACTC GGTATGTACT ACGAATCTTA CATACACAAA GCTCCAAGGA

4021 GGGTTGCATC AGTGACGGGC ATTGAATGGG TAACAGAAAC ATTATCAAAT CCGACCTCTT

4081 GTTATAACAT GTTTAGGATG AGTTGCCCAT TATTTAATCA ACTTCATGAT CTATTAGTTG

4141 ACTCATATGG TTTGAGAGCC ACTCGAGATA TGTCAACAGT GGAGGCCTTA GGAATGTTCC

4201 TATGGATATT AGGTGCACCG CAGTCACTTA GGCAAGTTGA GGATCGATTT GTGAGGTCGT

4261 TGGAGACAAT AAGCCGTACG TTCGACAAAG TGTTAGGCAC TGTTCTTAAG CTAGCAGTAC

4321 ATAATATTAG GCCACAGGAC CCTGAATTTA AGACGGTGCA CAAGAGATTA CTCAACCCTC

4381 GGTTTGCTCC GTATTTCAAC AACTGTATTG GAGCTATTGA TGGGACACAC GTTCCAGTCG

4441 TGGTGCCAAG TGAAAAGGTC ATGCAATATA CAAACAGGCA CGGGTATACC TCACAGAATG

4501 TGTTAGCTAT TTGTGACTTC GACATGAGGT TTACATCTGT TGTTAGTGGA TGGCCAGGAT

4561 CGGTCCATGA TATGAGAGTG TTCAGTGATG CCATAGAAAA ATACGGTGAC AAGTTTCCAC

4621 ATCCTCCTAC AGGTACACTG TTTCTATATG ATAGTTTTTT TGCCTATATG TTCTCTAGTT

4681 TGTTTTTACC TAACAAGTTT GCCTTTTGTT TGTAGGCAAG TTTTACCTTG TTGATTCGGG

4741 GTATCCAAAC CGTCCCGGTT ACCTATCACC TTACAAGGGT ACGAAGTACC ATCTACCGGA

4801 GTTTCGTAAT GGTCCAATGC CCAGAGGTAT GCAAGAGACC TTTAATTATG CACATTCATC

4861 CCTTAGAAAT GTTATCGAGA GGTCATTCGG AGTTTTGAAG ATGAAGTGGA GGATACTGAT

4921 GGGTATACCA AGTTTTCCAA TGCACAAGCA AAGCAAAATT ATTGTGGCTT GCATGGCAAT

4981 TCACAATTTT ATCCGAGAGA ATAGTGTTGC CGATAGGGAA TTTGATTTGT ACGATTGTGA

5041 TGAAAATGGT GTCCCAATGC CCGGAACTTC AAACCGCGGA GGAGGTGAGA CAAGTACCCA

5101 AGTAGAAGAA GAAGATAGCA ACATGAATGC ATTTCGAGAT GAAATAGCTC ATGCCTTGTA

5161 CAATAGGTCT AGATAAATTG ATTTCAATGT TGTAATGATA ATGTATTTGT AGTTTAATTT

5221 TGGAGTTGTT GGACATTGTA ATAGACATAA AATTCCCTTC TTATGCACTG GTGTATGACT

5281 GAAAGAGAAA GAGAAGAAGA GAGTGAAAGA GAAAGAGAAG AAGAGAGTGG AGAAGAAACA

5341 AGAAGAAAGG CATAGCGCTT TGCATGCATG CAGTGCAAGG AGGAGAACGG AGGGCACGGC

5401 ACAGCACCGC ACCGCTATGC ACGCATGCAT GCAAGCAGCG CCAGCGAGTG GAGCCAGCGC

5461 AGCACGCATG CATAGCAAGG GGCGCCAGCG AGTGGAGCCA GCGCAGCATG CATGCATGCA

5521 AGGGGCGCCA GCGAGTGGGG CCAGCGCATG CATGCGCGCG GGCGGGAGGG GCAGCGCGCG

5581 CGGGCGGGAG GACAAAAGCA GGGGTATGGT AGTCATTTTC ACTATTAGGT GCTAAATTTT

5641 AGCCTCCCAT CCAAACACCC ATGGGTGCTA AATTTTAGCC TCTTTTTTTG AGTGGGCTAA

5701 ACTTTAGCTC AAGGCTAAAT TTTAGCCCCT ACTCCCAAAC AGGCCAAGTT AGGTTAATTT

5761 TGTTTCATGG AAACTGTTTA GTACAACTCA TGTTCCTCAT TTCGATGCAT GCAACCACAC

5821 ACTACTGGAA ATATAAAATC TTCAGTTCGT ACTCACAAGT CAGAAGCACC CCCGGATAAT

5881 CCGAATCTGA CCTCCAGTGG TAACTGTAGC ACAAGTCCAC ACTTAGATCC GTCCGGAGGG

5941 ATAATAAATC TGTAATTACT TTTTGACTTG TTGTAAATAT ATTTGTCATT TTCATGCAGT

6001 CAAATCCAAA TTTAAATCAC CTGAATCACC CATGGTTCAA ACATCGAACC CACTGTTTGT

6061 TACTGATGAC TCAGAAAGTG CATCCACCTT TCCACCGCAG CTAGCTAGCC CTCCACGCTG

6121 GTCACCGGC**A TG**GCGGCTCT GGCCACTTCC CAGCTCGTCA CCACCCGCGC CGGCTTCGGC

6181 CTCGGCGACG CCTCCTCCTC CATGTTCCGC CCCGGCGTCC AGGGCCTCAG GGGCTCCCGG

6241 GCCTCCTCCC CGGCGGCCAC GCTCAGCGTG CGGACCAGCG CGCGCGCCGC GCCCAGGCAG

6301 CAGCACCGCC GGGCGCAGCG CGGCGCCAGG TTCCCCTCCC TCGTCGTCTG CGCCACCGGC

6361 GCCGGCATGA ACGTCGTCTT CGTCGGCGCC GAGATGGCGC CTTGGAGCAA GACCGGCGGA

6421 CTCGGCGACG TCCTCGGCGG CCTCCCGCCG GCCATGGCCG TAAGTCCCAT CGATCGATCA

6481 TCTCTTCCCT TCTCTTGTCG TGGACTCAAA GCTCATCGTG TCTTGTTGTC GGATGTACGT

6541 GATGGCGTGC ATCGTGTCAT TGGTGCATGC GTGTGTCATG TGTGTGACGT GCACGTGTGT

6601 TGGCTTGTGC AGGCGAATGG GCACCGGGTC ATGGTCATCT CCCCCCGCTA CGACCAGTAC

6661 AAGGACGCCT GGGACACCAG CGTCGTCTCC GAGGTACTTG ACTGCTCACA CCTTCAGAAG

6721 AGAATGAAAC TGTCCATCTC TCGCTGACAA AAGATCTGAA CGTTTGTTGT GCGTCTCCAT

6781 GCAGATCAAG GTGGGAGACA GGTACGAGAG GGTGAGGTTC TTCCACTGCT ACAAGCGCGG

6841 AGTCGACCGC GTGTTCATCG ACCACCCGTC CTTCCTGGAG AGGGTGAGAT TTCATTCATC

6901 GTCCCTTCTT GTTGTTGTTG TTTCTGACCA TGGCAGGTGA AAAGAAATAC TACCATCCGA

6961 TTGGCAGTTT CAGTGAACCT TTTTCTTTTG GACGGTGATT TCTTTCAGGT TTGGGGAAAG

7021 ACTGGTGAGA AGATCTACGG GCCAGACGCT GGAGTGGATT ACAAGGACAA CCAGCTGCGT

7081 TTCAGCCTTC TTTGCCAGGT CAATCACTAG CGATTGTAAT ACTGAATTCG GATCGGTCTG

7141 TATGTACTGC CATTTTTGTT GTGCTAACTC TTGGAAATGT TCGAATGAAC GCTACTGAAT

7201 GAATGCAGGC AGCACTTGAA GCTCCTAGGA TTCTGAGCCT CAACAACAAC CCTTACTTCT

7261 CAGGACCGTA CGGTAAGAAC ATAGTAGTAG TTGTGCTCTC TGATCTGCAT GAACTTGATA

7321 GTTCTGCACC AAGAATGGTC CATCAGACAT TCTTTCAGAC TGCAATTTCA CACCGATTAC

7381 TCTGCATTCA TCCAGGGGAG GACGTCGTGT TCGTCTGCAA CGACTGGCAC ACCGGCCCTC

7441 TGTCGAGCTA CCTCAAGAGC AACTACCAGT CCAACGGCAT CTACAGAAAC GCCAAGGTTC

7501 AGTTCTTCTC TTGGAAATCG TTTGATGTTC GTGTACACCA TTTCGTTCTG AAACTGACCG

7561 TCTGCCCCCC GGCACGTCCT AGACCGCTTT CTGCATCCAC AACATCTCCT ACCAGGGCCG

7621 GTTCGCATTC TCGGACTACC CGGAGCTGAA CCTCCCTGAG AGATTCAGAT CATCCTTCGA

7681 TTTCATCGAC GGGTACGTGC AGACTTGCAA TCTTTCTTTC AACTGTTTTT CATCATATCG

7741 ATACTGTTGG GGTCGATATA AGTTGACGTG ATCAATACAT AGCTCACTGT CCACTCTGGT

7801 TTGCAGCTAC GAGAAGCCTG TGGAGGGCAG GAAGATCAAC TGGATGAAGG CCGGGATCAT

7861 TGAAGCCGAC AGGGTCCTGA CCGTGAGCCC CTACTACGCC GAGGAGCTCA TCTCCGGCAT

7921 CGCCAGGGGC TGCGAGCTCG ACAACATCAT GCGCCTCACC GGCATCACCG GTATCGTCAA

7981 CGGCATGGAC GTCAGCGAGT GGGACCCCAG CAAGGACAAG TACATCGCCA CCAAGTACGA

8041 CGTGTCAACG GTGAGCTCTC GCCAAATTGT CTGTTTGAAT TGTTGTGCAA GTGTTCAGTG

8101 CGTCGGACAG ACCCTGACAG CATGACCCAC CATTTTCAGG CCATTGCGGC CAAGGCGCTC

8161 AACAAGGAGG CGCTGCAGGC CGCGGCCGGG CTCCCGGTGG ACCGGAAGAT CCCGCTGGTG

8221 GCGTTCGTCG GCAGGCTGGA GGAGCAGAAG GGCCCCGACG TCATGGCTGC CGCCATCCCG

8281 CAGCTCATGG AGGAGGATGT CCAGATCGTC CTTCTGGTAC GTAGCTACTA CAGCTTTACA

8341 TCCGAGTGCT GTGGCTATAC ATTCAGATTT CAGAGCAGTG TGGTTGATTT GATTTGATCG

8401 CGGTGAACAA TGAATGGTGC AGGGCACTGG GAAGAAGAAG TTCGAGCGCA TGCTGATGAG

8461 CGCGGAGGAG AAGTACCCCG ACAAGGTGCG CGCCGTGGTG AAGTTCAACG CGGCGCTGGC

8521 GCACCACATC ATGGCCGGCG CCGACCTGCT CGCCGTCACC AGCCGCTTCG AGCCCTGCGG

8581 CCTCATCCAG CTGCAGGGGA TGCGATACGG CACGGTACTT TCACTACTAC TGCTGCGCGC

8641 AACTCCCCCT ACTGAACTCT TTCTTGCCTT GCCAATGGCA TCCTGAAAAC GATGGACCGT

8701 GCCTCGACTG ATCGATCTCT TGCAATTGCT CTTGCAGCCC TGCGTGTGCG CGTCCACCGG

8761 CGGGCTCGTC GACACCGTCA TCGAAGGCAA GACCGGATTC CACATGGGCC GCCTCAGCGT

8821 CGACGTAAGG CTCTTTTCTT TTGTTTTCTT ATTGGTTGCA ATTGAAGATT CAGCGTCGAC

8881 GTAGGGCTCT TTCTTTTCTT TGTTGCAATT GTAGATTCAG GTAACTTCTT CTGTATATCA

8941 ATGGTGTAAC TAAACCAACG CCTCCTTCCC AGTGTAAGGT CGTGGAGCCG GCCGACGTGC

9001 AGAAGGTGGC GAGCACCCTG AAGCGCGCCA TCAAGGTCGT CGGCACGCCG GCGTACGAGG

9061 AGATGGTCAG GAACTGCATG ATCCAGGACC TCTCCTGGAA GGTACGTTAC GTCAGAGCAG

9121 AACAATTAAG CAAAACATCT GCAGCTTAGA CGATCACATT TGTATGCTCG TGTTGGTGAT

9181 GGTGGTTTGA CTGACAATGC TGCATCTGTA CGCGTACGTG CAGGGCCCTG CCAAGAACTG

9241 GGAGAACGTT CTGCTGAGCC TGGGCGTCGC CGGCAGCCAG CCGGGGATCG AAGGCGAGGA

9301 GATCGCGCCG CTCGCCAAGG AGAACGTGGC CGCTCCC**TGA** AGGCGGCCAA GATGATCCAG

9361 TGAACACCTA CATAGTATAT AGTTGCTTGT GGTAGTGTAT TGTAGTGGCC AGCGGCATAT

9421 ATGACCTAAT AAGTATGAAC TATATATATA CACTATATAT TGCGAGT

**Figure S5 The sequence information for the *waxy-slx* locus.** There are 9467 bp nucleotide bases including 14 exons, 13 introns and a *TSI-2* transposon insertion. The gray-labeled nucleotides indicate exons. The unlabeled nucleotides indicate introns. The dark blue-labeled nucleotides indicate the *TSI-2* transposon, which is inserted in Intron 1. The underlined and bold nucleotides in red color, ‘ATG’ and ‘TGA’, indicate initiation codon and stop codon, respectively. The sequence has been sent to NCBI with accession number KF372879.
